# Influence of Spider Silk Protein Structure on Mechanical and Biological Properties for Energetic Material Detection

**DOI:** 10.3390/molecules29051025

**Published:** 2024-02-27

**Authors:** Xinying Peng, Zhiyong Liu, Junhong Gao, Yuhao Zhang, Hong Wang, Cunzhi Li, Xiaoqiang Lv, Yongchao Gao, Hui Deng, Bin Zhao, Ting Gao, Huan Li

**Affiliations:** 1 Toxicology Research Center, Institute for Hygiene of Ordnance Industry, NO. 12 Zhangbadong Road, Yanta District, Xi’an 710065, Chinakeyan6_521@163.com (Z.L.);; 2 Xi’an Key Laboratory of Toxicology and Biological Effects, NO. 12 Zhangbadong Road, Yanta District, Xi’an 710065, China

**Keywords:** spider silk, structure, biomaterials, biomedicine, structural biology

## Abstract

Spider silk protein, renowned for its excellent mechanical properties, biodegradability, chemical stability, and low immune and inflammatory response activation, consists of a core domain with a repeat sequence and non-repeating sequences at the N-terminal and C-terminal. In this review, we focus on the relationship between the silk structure and its mechanical properties, exploring the potential applications of spider silk materials in the detection of energetic materials.

## 1. Introduction

**Figure 1:**
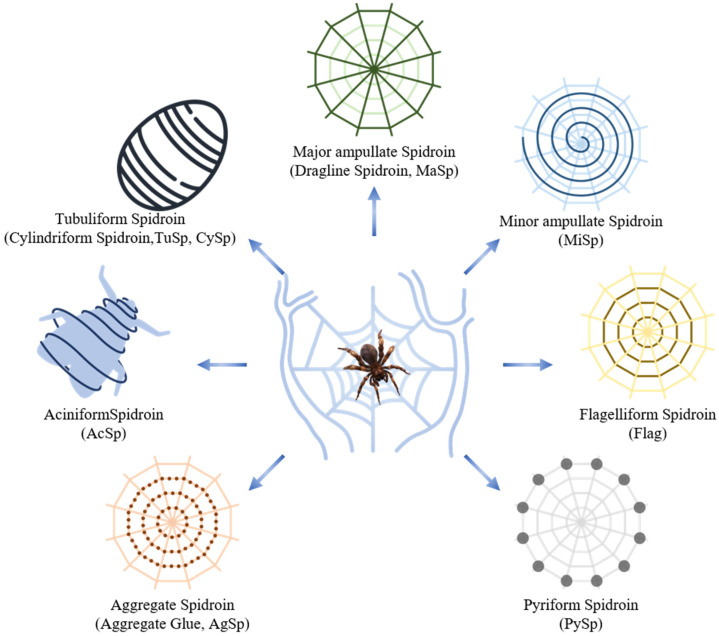
Schematic overview of silk produced by spider [9].

Natural spider silk fibers, having evolved over 400 million years, serve crucial functions in feeding, reproduction, and survival [1]. With a remarkable diversity of over 45,000 species, spiders exhibit the ability to produce task-specific silks with diverse mechanical properties originating from their unique silk glands [2,3]. Several types of spidroins, with different structures, determine their specific physical properties corresponding to their biological roles (Figure 1). Major ampullate silk (MaSp) (dragline silk) and minor ampullate silk (MiSp), known for their high strength, together with flagelliform silk (Flag), known for its high elasticity, contribute to the intricate production of a spider’s orb web, facilitating both its formation and effective capture of prey insects [4]. Aggregate silk (AgSp) (aggregate glue) provides spider silk with its renowned stickiness [5]. Tubuliform silk (TuSp) (cylindriform spidroin (CySp)) is the main constituent used by female spiders to manufacture egg cases [6]. Aciniform silk (AcSp) is applied for wrapping prey and protecting the egg sac from predators and environmental fluctuations, such as temperature and humidity changes [7]. Pyriform silk (PySp) is utilized in creating the attachment disc, enabling the web to adhere to different surfaces [8].

Exhibiting an intriguing blend of light weight, high strength, and exceptional elasticity, spider silk stands out as a stellar biomaterial. Its mechanical properties are on par with the best synthetic fibers manufactured by state-of-the-art technology. Its low toxicity and immunogenicity, coupled with its slow biodegradability and an apparent propensity for cell adhesion and growth, make it a compelling selection for biomedical applications [10,11]. In addition, silk threads also demonstrate interesting properties, featuring torsional memory, supercontraction, and self-healing [12,13,14].

Spider silk has been used by humans since ancient times. During the Middle Ages, and even as far back as the ancient Greek and Roman cultures, spider webs were employed for wound dressing [15]. The first pair of gloves and stockings made of spider silk were displayed at the Paris Academy of Sciences in 1710. Another pair of stockings was made by an American [16]. During the past years, dragline silk has been the most researched object of all the spider silks, while studies on minor ampullate silk, flagelliform silk, pyriform silk, aciniform silk, aggregate glue, and tubuliform silk have all been reported [3,9,10,11,12]. Nowadays, recombinant spider silk proteins have gained widespread employment in various fields, including biomedical, cosmetic, and technical applications.

However, owing to the territorial and cannibalistic nature of spiders and the challenge of generating substantial quantities of silk proteins at an economical cost, spider farming has been deemed impractical. Consequently, the best alternative is the biotechnological production of spider silk proteins. This process comprises two strategies: One involves the use of prokaryotic hosts, like *E. coli*, leveraging the well-established techniques of cell disruption and protein purification [17,18,19]. The disadvantage of prokaryotic expression systems is their inherent limitation in the size of synthetic genes. Another approach employs eukaryotic hosts, such as yeast, *Bombyx mori*, transgenic plants, bovine mammary epithelial alveolar cells, etc., which produce recombinant proteins most closely resembling natural silks [20,21,22,23,24,25,26,27,28]. Unlike the above-mentioned genetic engineering methods, chemical synthesis only replaces some regions of the silk protein with organic polymers [28]. However, the mechanical properties of the new fiber material are worse than those of natural silk.

In this review article, our focus is on how the silk structure determines its mechanical properties and its potential applications in detecting energetic materials. The repetitive domain is important for the mechanical properties of spider silk. Each repeat consists of crystalline stretches and amorphous areas, which collectively influence the strength and elasticity of the spider silk. The assembly of spidroins is controlled by non-repetitive domains, and some spider silk proteins feature a linker region bridging the terminal and repetitive regions of the silk protein. The structure and influence of these regions have yet to be discussed. Spider glues, microscopic glycoprotein nodules on capture silk that do not stick to each other, contribute to the stickiness of spider silk. Apart from the primary structure of spider silk, spinning techniques also have an important influence on the silk properties. Different forms of silk proteins have unique mechanical properties, rendering them suitable for various applications in different areas. Recombinant spider silks have found applications in diverse areas, particularly as exceptional candidates for biomedical applications, including tissue engineering, as well as in functionalized textiles, electronic engineering, cosmetics, etc. In response to the growing need for high-performance and eco-friendly materials in the sensor industry, it is essential to engineer green sensors capable of identifying explosive vapors at ambient temperature. Spider silk protein, which has shown promise in bioelectronic materials, could function as an eco-compatible sensory material for detecting the vapors of standard nitroaromatic explosives.

## 2. Relationship between Structural and Mechanical Properties of Spider Silk Proteins and Their Structural Benefits in Biosensor Applications 

### 2.1. Structure of Spider Silk Proteins

**Figure 2:**
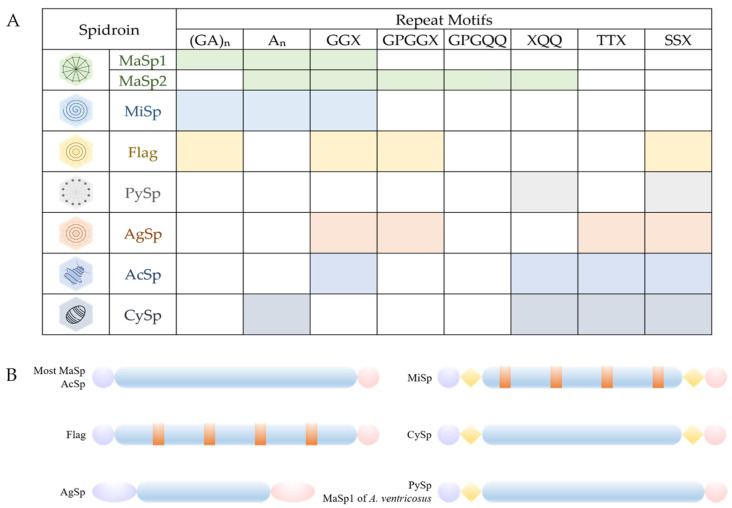
Structural motifs and correlated secondary structures of spidroins [36]. (**A**) The variety of motifs in the repetitive domains of different spidroins directly reflect their mechanical properties, allowing them to perform different tasks. A: alanine, G: glycine, P: proline, Q: glutamine, S: serine, T: threonine; and X: one of tyrosine (Y); leucine (L); alanine, serine, and arginine (R); valine (V); or glutamine (Q). (**B**) Spider silk proteins (spidroins) are mainly composed of a repetitive domain (blue) flanked by the non-repetitive and highly conserved N-terminal (purple) and C-terminal (pink). Some specific types of spidroins have a linker region (yellow) and spacers (orange) in addition.

Spider silk proteins exhibit a broad molecular-weight range, spanning from 70 to 700 kDa. They have a specific amino acid composition, which significantly deviates from that of structural globular proteins (Figure 2, Table 1). Structurally, spider silk proteins involve three distinct domains: a repetitive central core domain, organized with highly repetitive crystalline regions periodically interrupted by less repetitive crystalline peptides, and the conserved amino-terminal (N-terminal) and carboxy-terminal (C-terminal) domains (Figure 2B) [29,30,31]. All native spider silk proteins exhibit remarkably high levels of alanine, glycine, serine, and glutamine, while the contents of charged acidic and basic amino acids are relatively low. The repetitive regions of spider silk proteins contain sequence motifs, such as polyalanine stretches and glycine-rich areas [31], leading to different secondary structures that vary based on the spider species, the silk type, and the solid state of the silk fibers (Figure 2A, Table 1) [32,33].

Most silk proteins, including major ampullate, minor ampullate, and flagelliform silks, consist of short repeated sequence motifs in their repetitive regions. In contrast, aciniform and tubuliform proteins possess long and complex repeats without obvious simple internal motifs. The core domain of pyriform silk is composed of six repeated α-helix globular domains, each flanked by two disordered regions [34]. Regions of intrinsic disorder link the helical bundles, creating a beads-on-a-string architecture [34]. While the core domains differ, the N-terminal and C-terminal domains remain highly conserved across the silk types (Figure 2B). Furthermore, the semi-crystalline phase, along with the crystalline and amorphous phases, play a crucial role in the mechanical properties of spider silk fibers [35]. However, limited insights have been gained into the functions of each domain in PySps protein forms that utilize protein as cement in their attachment disks.

**Table 1:** Molecular weight, mainly containing amino acids and motifs, of each type of spider silk protein.

Spider Silk	Molecular Weight	Main Amino Acids	Main Motifs of Repeat Domain	References
MaSp	>250	Alanine Glycine	polyA or A_n_ polyGA or (GA)_n_ GGX GPGXX	[37,38]
MiSp	>250	Alanine Glycine	polyA polyGA GGX Spacer N-linker	[37,39,40]
Flag	>250	Alanine Glycine	polyGA GGX GPGXX Spacer	[37]
PySp	200–400	Proline Glutamine	polyPX or (PX)_n_ QQ-containing regions N-linker	[8,37,41]
AgSp	450–1400	Glutamine	Distinct repeat motifs Q-rich regions	[37]
AcSp	300–500	/	Long and complex motifs	[37]
CySp	180	Alanine Glutamine Serine	polyA QQ-containing regions S-rich motifs Linkers	[37,42]

Spider silk fibers produced in the silk gland exhibit a combination of spidroins with different structures. For instance, a silk fiber produced by the major ampullate gland is composed of two main proteins: the highly ordered hydrophobic major ampullate spidroin 1 (MaSp1), and the less-ordered hydrophilic major ampullate spidroin 2 (MaSp2) [43]. Other major ampullate spidroins are also found in specific spider species, which play important roles in their extraordinary mechanical properties. Major ampullate spidroin 3 (MaSp3), recently identified in the Araneus genus, is hypothesized to contribute to silk formation during the spinning process, although the precise relationship between its protein sequence and properties remains unclear [36]. Moreover, the discovery of major ampullate spidroins 4 (MaSp4) and 5 (MaSp5) in Darwin’s bark spider (*Caerostris darwini*) potentially explains the Darwini spidroin’s extraordinary toughness [44]. This suggests that several types of spidroins can be produced by a single spider species. The spidroin number in the velvet spider (*Stegodyphus mimosarum*) is 19, and in *Nephila clavipes*, the number is 28 [41].

### 2.2. Relationship between Different Functional Domains of Spider Silk Proteins and Their Mechanical Properties

Spider silk, recognized for its exceptional mechanical properties, which surpass those of high-performance manufactured materials like Kevlar and carbon fibers, also exhibits remarkable optical characteristics, such as nearly perfect transparency in the visible spectrum [45]. The structure of the silk fiber determines its mechanical properties. Through the exploration of recombinant protein block copolymers, we have obtained a crucial understanding that resolves essential problems in polymer science, including the thermophysical traits, self-assembly processes, and sequence–structure relationships of silk protein polymers. These insights have further inspired novel applications in biomedical engineering, such as gene transfer therapy and drug delivery [46,47,48].

#### 2.2.1. Repetitive Domains Mostly Influence Mechanical Properties of Spidroins

The crystalline domains play a dominant role in biomechanical studies of spider silk, with a crystal volume fraction ranging from 30% to 40%, depending on the spider silk species and measurement technique [49]. These domains are composed of polyA and polyGA repeat motifs, forming β-sheet crystalline domains with dimensions typically ranging between 1 and 10 nm during fiber formation, laying the groundwork for the tensile strength and stiffness of the silk fiber [31,50,51,52,53]. The nucleation and fibril growth are affected by the formation of β-sheets, with each β-sheet crystalline structure containing 6–12 alanine residues. Stable β-sheet nanocrystals result from the antiparallel alignment and stacking of polyalanine domains along the direction of hydrogen bonding. The mechanical properties of spider silk may experience improvement by increasing the number of alanine residues. However, beyond six alanine residues, the improvement in the mechanical properties becomes less significant [54]. The significantly enhanced mechanical properties of β-sheet nanocrystals are attributed to the utilization of hydrogen bonds and the emergence of dissipative molecular stick–slip deformation, given that hydrogen bonds are among the weakest chemical bonds [55]. Furthermore, hydrogen bonds serve as an optimal choice for establishing stable and robust bonds between the nanocrystalline and amorphous domains in silk [35]. Unlike MaSp1 and MaSp2, both MaSp3 and MaSp4 lack typical polyA motifs, which are replaced by serine-rich motifs [56].

Glycine-rich (G-rich) areas are mainly generated by (GGX)_n_, (GPGXX)_n_ (X = Y, L, A, S, R, V, or Q, 43 ≤ n ≤ 63) and are thus responsible for elasticity and flexibility [31,51,57,58,59,60,61]. Special G-rich motifs, such as GPX, GGGX, GGX-GGX-GX, (GX)noligoA-(GX)_n_, GGX-GGX-GGGX, and (GX)_n_, are also found in minor ampullate silk, flagelliform silk, and aciniform silk [37]. Interestingly, GPGXX motifs are not iterated within each G-rich repeat, as is the case of MaSp2 [38]. The amorphous structural analysis reveals that the regions consist of different contents of α-helix, 31-helix, β-sheets, and β-turns, with fewer dense hydrogen bonds [33,62]. The outstanding elasticity of silk fiber is attributed to the β-turns generated by (GPGXX)_n_ repeat motifs, positioned adjacent to (GGX)_n_ motifs and spacer motifs that form helices and β-sheets [63,64]. These weakly oriented and unaggregated β-sheets in amorphous domains are the primary contributors to the strain stiffening observed in spider silks [65]. Nanoscale mechanical deformation simulations trace the initial suppleness of the spider silk matrix back to the rupture of hydrogen bonds within domains rich in glycine [62]. Recent research indicates an abundance of post-translational modifications, predominantly phosphorylation and deamidation, that occur in GGX motifs. These alterations are projected to influence the conformation of the spidroins and, by extension, the physical properties of the resultant protein [66]. Due to the difficulty of detecting the amorphous phase in diffraction experiments, investigations into the molecular mechanics of this phase are limited [67]. In addition to the investigation into proline concerning its distinct impacts on peptide backbone structures and its contribution to supercontraction, recent research has established a potential association between tyrosine residues and supercontraction. The repetitive region of MaSp4 lacks GGX motifs and instead features unique GPGPQ motifs, increasing the flexibility of the spidroins by incorporating more β-turn conformation content within the fibers [56].

**Figure 3:**
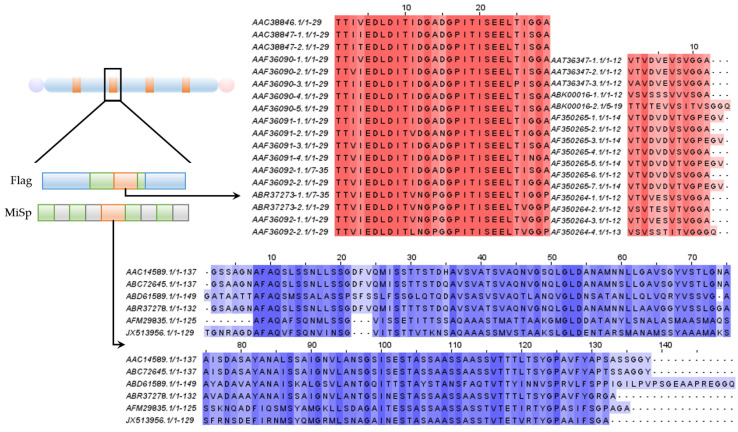
Molecular organization of spidroins with spacers. Spacer sequences were aligned using Jalview. Blue box in graphic represents GPGXX domain, green box represents GGX domain, gray box represents polyGA, and orange box represents spacer, the purple circle is N-terminal and pink circle is C-terminal. Sequences of spacer in MiSp are shaded in blue, and those in Flag are shaded in red. Gaps (-) indicate missing amino acid residues that have been inserted to align amino acid residues.

Several non-repetitive “spacer” regions are present within minor ampullate silk and flagelliform silk (Figure 3) [39,40]. The consensus repeat unit of the spacer consists of several GGX and GA/GGX/GPGGX domains, and these repetitive units alternate with the spacer regions. Sequences of spacers display near-identical patterns across different silk proteins [39]. Interestingly, the research on the spacer’s functions shows that the motif G’F’Y’ outperforms the motifs G’ (G’ is short for the motif (GGX)_n_), F’ (F’ is short for the spacer), and Y’ (Y’ is short for the motif (GPGGX)_n_) and their pairwise combinations in terms of toughness, strength, and moderate extensibility, while G’Y’ and G’F’ only present limited extensibility and decreased strength [64]. This result implies that the silk fiber’s strength is enhanced by the spacer motif, thanks to optimal molecular organization, β-sheet formation, and varied sequence complexity [64]. The unique property of the spacer allows the protein to safely revert to its original state post-elongation, without detriment to the silk’s mechanical or physical properties [60]. Furthermore, the spacer region might contribute to the β-sheet formation in silk fibers [60]. However, the exact structural nature of the spacer motif remains elusive, and additional research is required to pinpoint how the spacer motif influences the mechanical attributes of silk fibers.

In addition to the three regions mentioned above, there are some other specific regions in specific spidroins. The pyriform spidroin’s core repetitive region contains two distinct motifs: proline-rich motifs (PX)_n_ and QQ-containing motifs [8]. The (PX)_n_ motifs are characterized by their tendency to form random coils, in contrast to the QQ-containing regions, which are often shaped into α-helix or β-sheet structures [37]. Glutamine segments, also found in tubuliform silk, are thought to be involved in spidroin self-assembly [37]. X residues play a role in influencing the structure and function of dragline silk [37]. During periods of high metabolic costs for spiders, particularly when they need to produce a substantial amount of material for constructing the egg sac, there is an argument that the presence of serine-rich (S-rich) motifs in tubuliform spidroins serves to safeguard against the depletion of the serine content [42]. However, it is worth noting that S-rich motifs are linked to a slight decrease in the mechanical performance of tubuliform silk [42].

Silk comprises not only the well-recognized crystalline and amorphous phases but also a third phase referred to as the semi-crystalline phase. This phase can contain isolated β-sheets or other structures indicated by NMR, yet it appears disordered when analyzed via larger-scale X-ray diffraction [58,68]. It has been suggested that the semi-crystalline phase contributes significantly to the outstanding toughness observed in natural spider silk due to its superior energy absorption abilities [69]. The semi-crystalline phase develops at crystalline–amorphous interfaces, with the semi-crystal quality relying on the β-sheet nanocrystal length [35]. Yet, a comprehensive understanding of the features, stability, and composition of these semi-crystalline domains is still lacking.

The successful formation of spider silk-like copolymers relies not only on the β-sheet content but also significantly on the block repeats in the structure. An increase in the number of these repeats induces a transformation in the proteins from disordered structures into lamellar ones. The synthetic dragline-like proteins, produced through an *E. coli* expression system, demonstrated that reducing the number of repeats culminates in significantly shorter fibers. So far, construction with eight repetitions appears to be the minimum requirement for fiber polymerization, leading to flexible fibers that form free coils inside cells, featuring a constant diameter and chemical resistance [70]. Reducing this number to four leads to a loss of fiber flexibility and the emergence of unique “bird beak” structures, not observed in fibers with eight repeats [70]. The crystallization of the protein becomes easier as the number of hydrophobic block repeats increases [71]. This increase also boosts both the nucleation and growth rates, supporting the nucleation-growth mechanism typically seen in cross-β-fibril formation [72]. The assembly kinetics of spider silk fibers vary depending on the number of repeats [72].

Apart from their impact on the silk mechanical properties, repetitive sequences during silk self-assembly may also be designed for solubility and/or phase transitions; however, further studies have not been promoted [73].

#### 2.2.2. Non-Repetitive Terminal Domain Controls Spidroin Assembly

**Figure 4:**
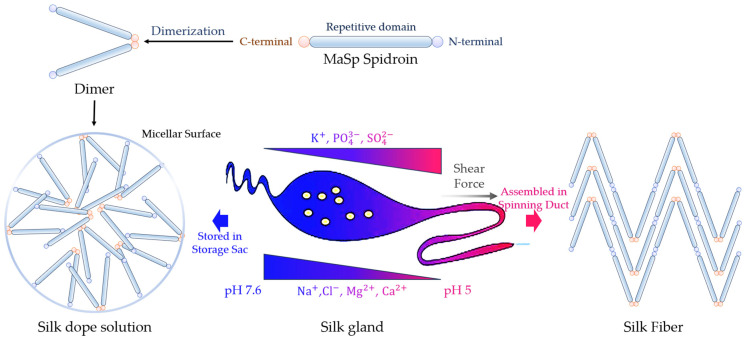
Schematic depictions of spider major ampullate silk gland and MaSp spidroin polypeptide chains [73]. Precursor spidroins, primarily adopting intrinsically disordered structures, can persist in a soluble state for long durations within the gland sac, serving as a concentrated liquid resource (silk dope). During the fiber creation process, spidroins traverse the serpentine spinning duct, undergoing extensive transformation. Conditions encountered in the duct include a pH shift (from neutral to acidic), an ion transition (from chaotropic to kosmotropic ions), dehydration, and the application of elongational and shear forces (due to the gradually narrowing duct structure). These collective circumstances trigger quick and precisely synchronized structural alterations in the distinct spidroin domains (N-terminal, repetitive domain, C-terminal), ultimately leading to the generation of large-scale silk fibers with a unique hierarchical arrangement.

Spider silk proteins’ terminal domains, in comparison to the repetitive domain with varying extended sequences among spider species, are small-sized (~110–130 residues) and exhibit high conservation across all types of spiders [74,75]. Both domains of MaSp show a homodimer of dipolar, antiparallel, five-helix bundle subunits, consisting of α-helical and loop elements [33,76]. They serve a critical function in regulating chain assembly, governed by changes in the ionic composition, mechanical stimuli, and pH during transit within the glands [18,33]. Moreover, the terminal domains are instrumental in preserving the solubility of spidroins at elevated concentrations (30–50% *w*/*v*) in the silk gland [9]. The pH-dependent dimerization is conserved between MaSp, MiSp, and Flag, although the amino acid residues mediating this process may differ [77]. CySp and AcSp have a different pH dependency mechanism than other silk proteins. CySp’s unique N-terminal amino acid sequence prevents full stabilization conformation at neutral pH and in low-salt conditions, while AcSp exists as a mixture of dimers, trimers, and larger oligomers under these conditions, unlike MaSp, which forms stable dimers [77,78]. However, further research on the terminals of CySp and AcSp is necessary, and the assembly mechanisms of AgSp and PySp assembly remain unclear. Thus, this review mainly focuses on the extensively discussed structure and function of the MaSp terminal domains in previous studies. While stored in the spider’s gland, silk proteins form micelle-like structures, with the terminal domains on the micelle surface preventing the non-specific aggregation of disordered, long, repetitive regions in solution and premature fiber formation [79,80]. The dimerization of proteins enhances the spinning-dope properties and fiber formation [81]. Notably, the presence of the C-terminal slightly destabilizes the structure of the N-terminal [82]. During silk assembling, there seems to be no interaction between the N- and C-terminal domains, simplifying the control of the spidroin assembly and minimizing unproductive side reactions (Figure 4) [82].

The C-terminal domain, instrumental in controlling the solubility, is of paramount importance in regulating the transition between the storage and assembly forms of silk proteins [79,83]. It also significantly influences the alignment of secondary structural features formed by the repetitive elements in the backbone of spider silk proteins, an essential critical factor in determining the mechanical properties of the resulting fiber [83]. Characterized by its dual thermo-sensitive behavior, the C-terminal domain predominantly undergoes a “gel-sol-gel” transition in response to varying temperatures [84]. Substituting the native C-terminal domain with non-native C-terminal domains from MaSp1 and MaSp2 results in fibers with reduced stiffness and strength, underscoring the significance of the native C-terminal domain [3]. In the examined silk types and species, an analysis of 19 families within the Araneae order demonstrates the consistent preservation of a pH-sensitive salt bridge, typically consisting of an arginine–glutamic acid pairing [85]. This notable conservation strongly supports the essential involvement of this characteristic in the accurate assembly of silk fibers.

Under conditions in the gland reservoir of a pH of 7 and 300 mM of NaCl, the N-terminal exists as stable monomers. However, in conditions resembling the spinning duct characterized by a lower pH (pH 5.5) and reduced NaCl concentrations, the N-terminal domain undergoes dimerization in an antiparallel orientation [86]. Its role includes enhancing the solubility of the spidroins during storage and facilitating self-assembly as the pH decreases along the spider’s silk extrusion duct, preventing premature aggregation [76]. The functionality of the N-terminal depends on the interchange between its monomeric and dimeric forms [87]. The dimerization of the N-terminal domain is pH-responsive [88]. With a drop in pH from 7 to 5, the N-terminal domain transforms its conformation into an antiparallel homodimer, a molecular detail that remains to be fully elucidated [89,90,91]. Acidification induces an instability in the C-terminal structural domain, prompting it to unravel into β-sheet formations. Remarkably, N-terminal dimers can transition into silk fibers at an exceedingly high pace (>1 m/s), all while being confined to the most distant part of the spinning ducts [90,92].

#### 2.2.3. Linker Regions Influence Silk Fiber Properties and Assembly

**Figure 5:**
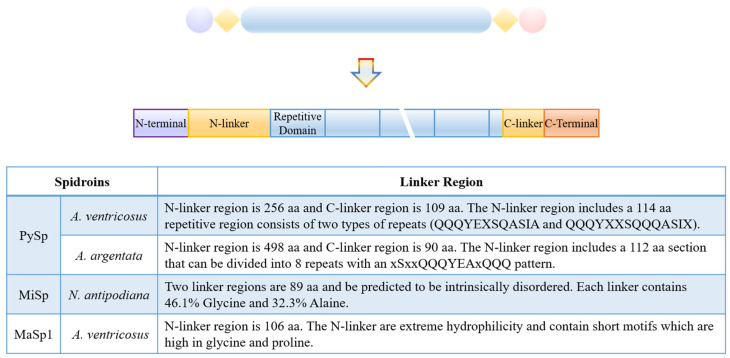
Molecular organization of spidroins with linkers.

Some spider silk proteins, such as MaSp1 and MaSp3 of *Araneus ventricosus*, pyriform spidroins, and minor spidroins, possess uniquely long and repetitive “linker” regions that bridge the terminal and repetitive regions of the silk protein (Figure 5) [39,93,94]. The linker sections vary in their amino acid counts, ranging from 83 to 174, and are characterized by a significant abundance of serine [39,94]. These “linkers” typically tend to be short in most spidroins, with pyriform spidroins being a notable exception, serving as transitional structures from the terminal regions to the medially repetitive region [41]. Each domain, including the terminal, repetitive, and linker domains, is characterized by a distinct stability and solubility, playing specific roles in ensuring the stable storage of silk protein fragments [94]. Due to their propensity for oligomerization, the linker regions and repetitive region in minor ampullate spidroins can initiate oligomerization through weak hydrophobic interactions, subsequently forming the core region of the oligomers [94]. Serving as a nucleation site, the linker domain facilitates the assembly of diverse molecules, while the terminal domains ensure their highly ordered arrangement under shear force [94].

Pyriform silk is distinguished by its long N-linkers and short repetitive region [95]. In each of these repeats, there is a more hydrophilic, glutamine-rich segment that contrasts with the rest of the repeat sequence [93]. Insights into the MaSp1 spidroin’s alternative splicing phenomena highlight the crucial role of the N-linker region in facilitating the self-assembly, stability, and mechanical properties [96]. The research also introduces an approach to devise recombinant spider silk proteins with superior mechanical properties. On the contrary, lacking a unique structure, the C-linker in pyriform silk marginally surpasses others in length [97].

Proteins with glutamine-rich regions alone have shown the ability to self-assemble into fibers, and their flexibility is enhanced with the addition of a proline-rich region [93]. It is hypothesized that the N-linker repeat region alone can independently form fibers, and incorporating flanking N-linker sequences might influence the self-assembly rate or mechanical attributes of the fibers [93]. However, the structure and influence of the linker region still need to be discussed. A deeper understanding of these mechanisms necessitates in-depth studies on the structures of the assembled forms [93,94].

#### 2.2.4. Spider Silk Glues and Viscidity of Silk Fiber

Spiders spin their capture threads in a distinctive manner, forming a central flagelliform fiber interspersed regularly with glue droplets. This configuration enables the spider to move freely within its web, ensuring that any prey making contact with the web encounters numerous nodules simultaneously, leading to effective entrapment [98]. The water-soluble silk glue, primarily produced by the aggregate gland, and considered one of the most effective biological glues, manifests as microscopic glycoprotein nodules that avoid mutual adhesion [99,100,101]. The extensive, rough endoplasmic reticulum forms small vacuoles, which are then synthesized into secretory vesicles, travel through the Golgi complex, and release aqueous glue through the mechanism of apocrine secretion into the lumen [101]. Unlike fibrous silk production, the Golgi complex plays an important role in glue production, involving either a condensing or packaging step [101]. The glycoprotein-rich glue tightly surrounds the twin internal axial fibers produced by the flagelliform gland.

In contrast to other spider silk proteins, silk glue is a viscoelastic, amorphous, wet material that responds to environmental conditions [102]. It is noteworthy that the glue droplets of cobweb weavers demonstrate greater firmness and resilience compared to those of most of their orb web-weaving counterparts [103]. The aqueous nature of the glue enables it to absorb moisture from the surrounding humid environment, thereby reducing the surface tension of spider silks and enhancing their distinctive elasticity [104].

The coding sequences of proteins in aqueous silk glue represent one of the largest groups of silk protein genes documented to date [102]. Predominantly, the sequence is composed of two distinct repeat motifs, measuring 387 bp and 339 bp, interspersed by glutamine-rich regions [102]. In contrast to other spider silk proteins, the glue proteins exhibit pentameric QPGSG repeats, bearing a striking resemblance to preserved modular elements in mammalian elastin, an elastomeric protein interacting with collagen [105]. The precise locations of galactose, mannose, glucosamine, caramel, glucose, N-acetylgalactosamine, γ-Aminobutyramide, N-acetyltaurine, choline, betaine, and ethionic acid are also identified within these nodules [99,106].

The glue droplets consist of three regions, incorporating these glycoproteins, low-molecular-mass compounds (LMMCs), and organic salts [107]. These droplets comprise a central opaque anchoring granule, a surrounding middle region of larger transparent glycoprotein, and an outermost fluid and viscous coating. The adhesion of a single glue droplet relies on a delicate balance between two factors: the interfacial adhesion and bulk dissipation. Proteins present in aggregate glues are characterized by higher levels of glycosylation and phosphorylation compared to proteins in other silk fibers. These modifications likely contribute to the stickiness of aggregate glue and may also enhance its cohesion. The glycoprotein core’s adhesion is influenced by the presence of low-molecular-mass compounds (LMMCs) in the surrounding aqueous layer. These LMMCs solvate the adhesive glycoprotein, imparting hygroscopic properties, softening the glue, and enhancing its adhesion. Additionally, salts play a direct role in facilitating adhesion by solvating the glycoproteins and increasing their mobility. However, the specific proteins comprising the cores of aggregate glue droplets remain unknown at present. The precise mechanism by which spiders assemble glue proteins into fibrous-like materials is a subject of considerable debate [105].

Glue drops function as a viscoelastic material that enhances adhesion through capillary forces [108]. The viscosity of the glue is believed to respond to humidity, aiding spiders in adapting to their local environments [109]. While high humidity typically inhibits adhesion, spider glues become stickier with increasing humidity levels [110]. Atmospheric water uptake acts as a plasticizer, enhancing glycoprotein plasticity rather than adhesion. Consequently, the viscosity decreases as the humidity increases [111,112]. Despite significant variation in the glue viscosity across different species, spanning five orders of magnitude in response to humidity, the viscosity at maximum adhesion remains remarkably consistent for each species [109]. The distinct humidity response exhibited by the glue components, distinct from the pristine glue, reinforces the hypothesis that the synergistic association among the spider glue constituents plays a crucial role in its adhesive functionality [113]. In addition to humidity, factors such as the strain rate, temperature, ultraviolet radiation B (UVB) radiation, and spider’s protein intake level also influence the adhesive properties of viscid spider silks [104].

### 2.3. Benefits of Spider Silk Structure When Silk Is Utilized in Biosensors

The interest in flexible sensing materials has increased in recent decades due to their technological potential. These materials provide stretchability, compressibility, twistability, bendability, and, in general, deformability to sensors [114]. Spider silk protein, especially the dragline silk protein, is one of the most attractive flexible materials that is applied in sensors, due to its excellent biological and mechanical abilities, light waveguide ability, and humidity sensitivity [115]. Their unique structures, which cause specific conformations and excellent properties, are the most important influencing factors for their utilizations in biosensors.

In addition to the outstanding mechanical and biological properties that we mentioned above, the structure of spider silk threads with round cross sections and, thus, good light-guiding properties, make them a candidate for use as optical fiber sensors [116,117]. In addition, spidroins are ideal humidity sensors because of their “supercontraction” properties when wetted or saturated in a high-relative-humidity (RH) atmosphere [118]. When the spidroins are laid in wet conditions, H_2_O molecules infiltrate the silk fiber and disrupt the hydrogen bonding of the amorphous region, resulting in the rearrangement of the configurations [119]. These rearrangements of the protein structures change the silk modulus and cause the reversible supercontraction phenomenon. Moreover, spider silk proteins act as natural pH-sensitive materials that can be integrated to develop pH sensors without further processing [120]. When in an acid or alkaline solution, the N- and C-terminals of the silk proteins react with hydrogen ions and hydroxide ions, and the hydrogen bonds are modified by the acid or base, resulting in the reversible denaturing of the spidroins [121]. These changes in the SDS protein structure lead to the change in the refractive index (RI), with the wavelength red-shifting when the pH increases and blue-shifting when the pH decreases [120].

Metal nanostructures as well as quantum dots and dyes can be easily attached to the surfaces of spider silk fibers without complicated surface treatment [122]. Reversible hydrogen bonds, holding the repetitive crystalline and amorphous regions together in the fiber, can be modified by different molecules, making the silk ideal for detecting modifying agents such as water molecules, acids, bases, etc., allowing for potential applications in silk-based biosensing [123]. Because even a 10 mm spider silk thread is composed of quantities of spidroin proteins, it is theoretically possible to detect a multitude of compounds with only a single silk thread with the benefits of high sensitivity and a quick response time.

Though the properties of spider silks utilized in biosensors should be further analyzed from the perspective of their molecular compositions and structures, the application of spider silk materials in the biosensing field has broad prospects. Moreover, with the continuous deepening of spider silk biosensing research, the mysterious veil over the structure and properties of spider silk protein is gradually being lifted.

## 3. Influence of Production and Spinning of Spider Silk Proteins on Their Properties and Their Influences in Spidroin Biosensing Research

### 3.1. Properties of Recombinant Spider Silk Do Not Compare with Those of Native Silk Fibers

The properties of synthetic spider silk, derived from recombinant processes, do not match those of native silk fibers. The challenges associated with commercial spider farming, such as the aggressive and cannibalistic nature of spiders and the difficulty in producing sufficient silk proteins cost-effectively, make it an unviable option [124]. Therefore, biotechnological production offers the best solution for generating silk proteins. However, the properties of recombinant spider silk proteins produced by various production strategies, especially their mechanical properties and water solubility, show significant deterioration compared to those of native silk.

Extensive exploration of recombinant spider silk production has focused on bacteria and other prokaryotes as potential host systems for producing spider silk proteins. These microorganisms offer advantages in terms of gene manipulation and metabolic engineering, making them cost-effective for fermentation production [125]. The utilization of *E. coli* for spider silk production has gained prominence in recent years (Table 2). To establish commercially viable production platforms, studies investigating the cost-effectiveness and environmental implications of manufacturing synthetic spider silk have been initiated [126]. However, the effectiveness of employing *E. coli* for producing spider silk proteins is hindered by the silk gene’s instability and the sluggish production rate, which further influence the mechanical properties as well as other properties of spidroins [19]. The obtained proteins may possess affinity domains, which may alter the protein properties, disrupt their function, and even cause protein cytotoxicity [127]. Furthermore, recombinant spider silk fibers with specific affinity domains (such as His-tag) are brittle and difficult to manipulate [128]. Moreover, recombinant spider silk proteins are generally insoluble in aqueous solutions, while natural spider silk proteins remain soluble in a concentrated state (approximately 50% *w*/*v*) within inorganic salt solutions in the silk glands [129]. To help dissolve the recombinant protein, a large number of organic solvents and additives proven to be hazardous to human health and the environment are used, the residues of which inevitably increase the cytotoxic effects in organisms [130]. Although several ways to obtain recombinant spider silk protein aqueous solutions have been researched in previous studies for biomaterial development, it is still very difficult to access high-molecular-weight, water-soluble spidroin proteins [129].

Employing eukaryotic hosts is an alternative approach to spider silk production, enabling the production of recombinant proteins closely resembling their natural counterparts (Table 3). Yeast, owing to its robust research background and unparalleled proficiency in expressing heterologous proteins, is extensively employed for producing spider silk proteins. Notably, proteins expressed in *Pichia pastoris* closely mimic the amino acid sequence and structure of native proteins, establishing it as a promising expression host for recombinant silk proteins. Numerous plants, such as tobacco, potato, and *Arabidopsis thaliana*, have also served as expression hosts for spider silk protein genes. While spider silk proteins expressed in animal hosts lack size restrictions and exhibit a low rate of gene recombination, challenges arise in efficiently purifying the protein due to organism aggregation. Moreover, the time-consuming nature and high costs are inherent issues in eukaryotic expression systems.

Chemical synthesis is also a potential method of spider silk production. In contrast to the aforementioned genetic engineering methods, chemical synthesis involves replacing regions of silk protein with organic polymers. Sogah’s discovery of a bioinspired synthetic material, consisting of poly(ethylene glycol) (PEG) and (AlaGly)2 segments, allows for self-assembly into nanostructures, mimicking *B. mori* silk’s solid-state properties [28]. However, the mechanical properties of the new fiber material fall significantly short of those found in natural silk.

An essential determinant in matching the mechanical properties of natural fibers is the high molecular weights of natural spider silk proteins. Consequently, a challenge in recombinant spider silk production lies in expressing silk proteins with molecular weights equivalent to those of natural silk proteins. Despite extensive efforts and encouraging progress, biomimetic approaches to fabricate native-like fibers with comparable mechanical resistance have seen limited success so far [134]. Full control over the fiber structure and mechanics remains incomplete, and the utilization of fusion or hybrid proteins frequently serves as an alternative route for the creation of innovative biomaterials.

### 3.2. Spinning Process Has Important Influence on Silk Properties

#### 3.2.1. Influences of Natural Spinning Process of Native Spider Silk Proteins on Silk Properties

The spider silk properties are greatly influenced not only by the primary structure of the silk, but also by the processing of the protein solution [134]. Spiders convert this aqueous solution into a solid fiber under ambient conditions [135]. The lack of a defined tertiary structure in the core domain of spider silk protein in the solution results in its transformation into insoluble cross-β fibrils after spinning [136,137]. Two models, the liquid crystal model and micellar model, describe the organization of this process. These models dictate that protein molecules establish specific structures in the solution, either forming liquid crystalline structures or generating micellar structures. This sequence formation lessens the fluid’s viscosity, grants it non-Newtonian traits, and prepares the proteins for subsequent structural changes, resulting in solidification.

In the spider gland, both the Na^+^ and Cl^–^ concentrations progressively decrease as the silk dope travels down the duct, while the K+ concentration rises [138]. The pH of the dope also falls from 6.9 to 6.3 during its passage through the duct [138]. During native spinning, shear forces, water segregation, the pH, and ion exchange control the structural transition from the spidroins’ storage state to the assembled state [139,140]. The transformation of cylindrical, aciniform, and piriform silk proteins from solution to solid-state fiber involves a change in structure from globular α-helical domains to mixed α-helix/β-sheet fibers. The mechanical properties of spider major ampullate silks significantly change with variations in the speed and temperature of the drawing. The re-alignment of protein chains in spider silk, facilitated by stretching, is achieved by restoring the network of hydrogen bonds [141]. Spider silk’s mechanical properties can be effectively and consistently modified through a simple technique called wet stretching [141]. The mechanical properties and supercontraction of spider silk could also be adjusted using reeling conditions [142]. Evidence is mounting that the upper structures in the hierarchy are not just shaped by the primary structure but are also notably influenced by the process of spinning itself. A thorough investigation of the silk-spinning process and its associated material properties could offer exceptional insights into the structure–property–function relationship of this extraordinary biomaterial.

#### 3.2.2. Artificial Spinning Technology of Recombinant Spider Silk Proteins and Their Properties

There are three main strategies for producing artificial spidroin fibers: wet spinning, dry spinning, and electrospinning. Silk fiber production predominantly relies on wet-spinning techniques [81]. Yet, this method faces challenges when applied to the spinning of bioinspired or regenerated fibers due to its complexity, the time and energy intensity, and the substantial amount of solvent required [143]. In contrast, dry-spun silk fibers could potentially exhibit superior toughness compared to their natural counterparts [144]. Notably, natural silk spinning is fundamentally an anisotropic or liquid crystal-based process, occurring under ambient physiological conditions without the need for additional immobilization or post-processing steps [139]. Electrospinning, which shares similarities with dry spinning, is another widely adopted fiber-spinning technology that allows for the creation of more delicate fibers [145]. Nevertheless, the fibers spun using these methods often display brittleness and inferior mechanical properties, necessitating complex post-processing treatments to yield practical fibers [143]. Critically, the current spinning techniques, including both wet and dry spinning, mainly aim to emulate the mechanical attributes of natural silks, neglecting to maintain the unique hierarchical structures that significantly contribute to the properties of these natural protein fibers [143].

By harnessing the inherent anisotropic dry-spinning features of natural spinning, it is possible to utilize a bioinspired spinning approach for producing polymorphic hierarchical silk fibers, and even for constructing intricate 2D and 3D structures [143]. The method of Straining Flow Spinning (SFS), employed for the spinning of fibroin fibers, has promising potential owing to its remarkable versatility and capability of spinning fibers, reaching lengths over 1000 m at present [146]. Previous research has shown that the number of crystalline and amorphous regions in the fiber, one of the most important influencing factors on the spidroin mechanical properties, can be tuned by changing the spinning speed and environmental conditions of the silk [147,148,149]. These attempts may facilitate the production of high-performance composites with superior mechanical characteristics.

However, the endeavor to produce the spinning of spider silk poses substantial technological hurdles. Not only does it involve the intricate process of synthesizing an insoluble fiber from an aqueous solution teeming with large proteins, but it also demands the ability to adjust the fiber’s tensile strength in split seconds [150,151]. Further complicating the challenge, most attempts to artificially manufacture spider silk fibers have relied on harsh conditions, such as toxic solvents or coagulants, or exposure to high-temperature processes [146]. Consequently, achieving the spinning of artificial silk fibers under mild conditions (aqueous dopes, low-toxicity coagulants, and ambient temperature) remains an ambitious scientific goal that has yet to be achieved [146].

### 3.3. Influence of Production and Spinning on Spidrion Biosensor Fabrication

As mentioned above, the production and spinning of spider silk proteins influence their mechanical and biological properties and water solubility. An adequate mechanical performance, biocompatibility, and water solubility are important to their long-term use in practical applications, especially applications in biomedicine [152]. In addition, spidroin materials can be functionalized by genetic engineering. The production of functionalized recombinant protein constructed at the DNA level combines the sequence encoding bioengineered spider silk, which is responsible for the biomaterial structure, with sequences encoding the polypeptides for functionalization [153]. Ideally, these bioengineered silk proteins would be fabricated as combinations of functionalized fibers and selective sensors to specific chemical compounds. Moreover, sensitivities to target molecules of spider silk proteins with known numbers of crystalline and amorphous regions can be custom-made, such as by tuning the percentage of the water-accessible β-sheets of the spidroin fibers, which can influence their humidity [154].

While natural spidroin proteins are suitable for concept exploration in laboratories, artificially fabricated spidroin silks need to upscaled to the industry level. There are still many barriers to overcome to fully exploit this highly promising material for the biosensing field. The attractive properties achieved in the production and spinning strategies influencing artificial spidroin materials will allow for the production of tailor-made, flexible biosensors. These would be the first building blocks towards the conception of a new generation of functionalized, biocompatible, selective sensitive devices.

## 4. Applications of Spider Silk Proteins

Spiders have been exceptional silk producers for the past 400 million years [1]. In recent decades, significant advancements have been made in comprehending the gene organization, assembly, and applications of spider silk. The production of silk is an environmentally friendly process, and the remarkable mechanical properties, lightness, biocompatibility (biosafety and biofunctionality), and biodegradation of these biomaterials contribute to their ability to produce customized primary structures for various novel functionalities. Due to these biocompatible and mechanical features, spider silk shows potential for the development of technical textiles, healthcare products, biomedical devices, biosensors, and more [155]. Spider silk proteins can assemble in various forms, providing versatility to meet diverse demands.

Regarding potential applications, spider silk proteins exhibit strong potential, especially in technical applications, like biosensing devices [156,157]. Biosensing is a multidisciplinary field that integrates biology, chemistry, electronics, and mechanics. Biosensors are devices that integrate components such as transduction mechanisms, biological recognition elements, and analytes. They are specifically engineered for the detection of particular analytes in environmental or healthcare contexts [158]. Upon interaction with the intended analyte, the biological recognition element yields a stimulus, which is then channeled and amplified through the transduction mechanism for analyte detection. Depending on the diverse transduction approaches, biosensors can be categorized into mass-based (piezoelectric and magnetoelectric) sensors, optical sensors, and electrochemical sensors (potentiometric, amperometric, impedimetric, conductometric, etc.) [159]. Over the years, wearable, flexible nanoengineered sensor materials, such as spider silk sensors, have seen a surge in development for the detection of a wide range of analytes with low detection limits [158].

Energetic materials (EMs), which are also known as high-energy-density materials (HEDMs), are special materials with high chemical energy [160]. Their applications in the aerospace, military, mining, and construction industries, and especially in explosives production, are widely recognized [161]. In the context of our dynamically changing global order, characterized by a rise in regional and ethnic conflicts, the persistent threat of terrorism and explosive attacks looms large. Exposure to explosives like nitroaromatic compounds (NACs) beyond safe levels poses various health risks, including eye and skin irritation, respiratory disorders, liver dysfunction, anemia, and others [162]. Therefore, detecting explosives has gained prominence as a crucial research focus, particularly within the realm of material science, for its essential role in ensuring homeland security, monitoring environmental health, and mitigating health hazards [163]. Many conventional detection techniques, such as gas chromatography–electron capture detection, surface-enhanced Raman spectroscopy, and energy-dispersive X-ray diffraction, are currently widely applied in the detection of explosive materials [164]. However, most of the devices used in these methods are expensive and not easy to carry, and their eco-incompatibility and sensitivity are not ideal. With the sensor market witnessing substantial growth, the demand for efficient, environmentally friendly materials for sensor development has reached new heights. Consequently, the quest for cost-effective, selective, and eco-friendly sensors for detecting explosive vapors at room temperature has become critical. The properties of biomaterials are increasingly being exploited in the approaches to fabricating physical and chemical sensors [165]. In particular, spider silk proteins have unique properties that can be used in sensors.

Singh et al. found that, upon exposure to methanol and chloroform vapors, a gold nanoparticle–spidroin bioconjugate material underwent a shift in the electrical transport of its nanoparticles [166]. This intriguing outcome initially suggests the feasibility of creating a biosensor predicated on spider silk. These sensors can detect small molecules (<900 Da), metal ions, macromolecules (>900 Da), entire cells, and specific environmental conditions [158]. Since then, spider silk proteins have been widely employed in various biosensors and have served as flexible substrates for biomedical applications in silicon nanomaterial-based electronic devices [123]. 

Spider silk, as an inherently moisture-sensitive material, requires no additional sensitization [118]. It is well suited for crafting humidity sensors, offering the benefits of reversibility, consistent repeatability, and eco-friendliness [167]. The remarkable supercontraction attribute of spider silk allows for the adjustment of its length with changes in relative humidity (RH), exhibiting considerable changes in high-humidity conditions [168]. This underscores the potential of spider silk to detect RH in high-humidity scenarios with great sensitivity, serving as a humidity alarm to shield storage spaces from moisture. Spider silk is also a naturally pH-sensitive material, needing no additional processing, and it can be integrated with optical fibers to construct pH sensors [114]. Its refractive index (RI) undergoes reversible adjustments based on the pH of the surrounding liquid environment, fluctuating within a reasonable range.

Except for their use as bio-recognition elements, spider silk materials are primarily used as stable substrates for attaching the recognition element, or as the basic structure upon which sensors are built [158]. Self-assembled spider silk proteins can self-assemble with inorganic particles like metals or quantum dots, offering a possible approach to the more effective functionalization of spider silk protein. For example, the interaction between the spider silk thread and FeCo layer strongly affects the magnetic behavior of hybrid material consisting of spider silk protein and the FeCo coating, resulting in changes in its magnetic signal when subjected to tensile strain [114]. Therefore, this spider silk composite material is suitable for magneto-electronic sensors. Apart from FeCo, a number of silk fibers containing inorganic materials, such as Ag, Au, Al, SiO_2_, Fe_2_O_3_, Fe_3_O_4_, CdTe, CdS, TiO_2_, and ZrO_2_, have appeared and are used as templates in synthesized biopolymers, resulting in new smart structural materials, all of which have potential in biosensor development [122,169,170,171,172,173,174,175,176,177]. In these biosensors, spider silk serves as a robust substrate to anchor the bio-recognition elements with minimal loss of their bioactivity. Therefore, their biocompatibilities allow for their use in living tissues without any immune reactions.

Spidroin materials can provide a platform upon which the sensor is fabricated and the recognition elements are immobilized. Their biocompatibility and bioactivity help maintain the activity of any biomolecule for longer durations, enabling the use of the biosensors in tissues without eliciting any immunological response. The use of easily obtainable flexible spider silk fibers as textile substrates in wearable sensing devices preserves the sensing functionalities [165]. Through recombinant production strategies, spider silk proteins with different molecular masses can be easily designed for specific aims. Their mechanical properties can be readily controlled as needed, and they can be effortlessly dissolved or mixed with other simple or complex molecules to form polymers and fusion proteins with the combined properties of all the molecules.

However, numerous challenges need to be addressed in order to overcome obstacles such as achieving native-like silk production, controlling the structure and properties of the biomaterials, and minimizing their ecological impact [134]. The limited number of biosensors developed using spider silk, in comparison to other biomaterials, could be linked to insufficient research and awareness. Hence, novel biomaterial designs should prioritize the intended purpose while considering factors like the appropriate mechanical, optical, biocompatibility, and biodegradability properties, as well as the ecological footprint throughout the lifecycle.

## 5. Conclusions and Outlook

Various spider species produce specific silk proteins in morphologically distinct glands. Since 1990, numerous silk genes have been identified for different silk proteins, accompanied by a plethora of physical studies. The spider silk protein comprises a core domain with a repeat sequence and non-repeating N-terminal and C-terminal sequences. The structures, properties, and functions of spider silk proteins depend on the arrangement of different amino acids. Spider silk fibers exhibit mechanical properties superior to the best synthetic fibers produced by modern technology, such as Kevlar and nylons. Additionally, spider silk exhibits excellent biological properties, making it attractive for biomedical applications. Apart from fibers, spider silk proteins possess exceptional versatility for processing in various colloidal forms, such as particles, foams, gels, films, sponges, porous systems, capsules, non-woven fiber mats, and emulsions. Their ability to be processed in aqueous solutions under ambient conditions, coupled with their favorable characteristics, makes them an excellent candidate for applications in electronic and photonic devices.

Researchers have shifted their attention from conventional substrates, like glass and silicon, to exploring the potential offered by biopolymer silk fibroin scaffolds, applicable for material development, device fabrication, and, notably, the detection of nitroaromatic explosives [178]. These sensors exhibit a significant change in resistivity when exposed to the vapors of nitroaromatic compounds, such as TNP and TNT. The similarity between spider silk and silk fibroin makes the use of spider silk materials and impedimetric sensing techniques an ideal choice for detecting explosive vapors, aligning with the ambitious goal of sustainability. It is, however, disappointing that spider silk materials have not been actively utilized in the field of explosives detection. Exploring this aspect could prove to be a significant avenue for future research and development.

## Figures and Tables

**Table 2:** Production of spider silk proteins in prokaryote host.

Protein	Protein Weight (kDa)	Production	Reference
MaSp2	45	10 mg/g	[131]
MaSp1	284.9	2.7 g/L	[18]
MaSp2	201	3.6 g/L	[124]
PySp1	47.9	30–60 mg/L	[132]

**Table 3:** Production of spider silk proteins in eukaryotic host.

Protein	Host	Production	Reference
DP-1B (MaSp)	Pichia pastoris	1 g/L	[20]
Z-4RepCT (fusion protein)	Pichia pastoris	/	[133]
Flagelliform silk	Bombyx mori	0.08 mg/flask (2 × 10^6^ cells, 6 mL)	[21]
MaSp 1	Nicotiana tabacum	0.5% TSP	[23]
DP1B (MaSp)	Arabidopsis	0.34% TSP (64 kDa) 0.03% TSP (127 kDa)	[24]
MaSp1	Nicotiana tabacum	0.7% TSP	[25]
MaSp2	Nicotiana tabacum	1.9% TSP	[25]
ADF-3 (MaSp)	Goat mammary epithelial cell	2.8–8.3%	[26,27]
ADF-3 (MaSp)	Bovine mammary epithelial alveolar cells	10–28%	[26,27]

## Data Availability

Not applicable.
